# Quick and improved immune responses to inactivated H9N2 avian influenza vaccine by purified active fraction of *Albizia julibrissin* saponins

**DOI:** 10.1186/s12917-020-02648-1

**Published:** 2020-11-07

**Authors:** Hongxiang Sun, Liyan Fei, Binnian Zhu, Minghua Shi

**Affiliations:** Key Laboratory of Animal Virology of Ministry of Agriculture, College of Animal Sciences, Zhejiang University, Hangzhou, 310058 China

**Keywords:** *Albizia julibrissin* saponin, Adjuvant, H9N2 avian influenza vaccine, Immune response

## Abstract

**Background:**

H9N2 Low pathogenic avian influenza virus (LPAIV) raises public health concerns and its eradication in poultry becomes even more important in preventing influenza. AJSAF is a purified active saponin fraction from the stem bark of *Albizzia julibrissin*. In this study, AJSAF was evaluated for the adjuvant potentials on immune responses to inactivated H9N2 avian influenza virus vaccine (IH9V) in mice and chicken in comparison with commercially oil-adjuvant.

**Results:**

AJSAF significantly induced faster and higher H9 subtype avian influenza virus antigen (H9–Ag)-specific IgG, IgG1, IgG2a and IgG2b antibody titers in mice and haemagglutination inhibition (HI) and IgY antibody levels in chicken immunized with IH9V. AJSAF also markedly promoted Con A-, LPS- and H9–Ag-stimulated splenocyte proliferation and natural killer cell activity. Furthermore, AJSAF significantly induced the production of both Th1 (IL-2 and IFN-γ) and Th2 (IL-10) cytokines, and up-regulated the mRNA expression levels of Th1 and Th2 cytokines and transcription factors in splenocytes from the IH9V-immunized mice. Although oil-formulated inactivated H9N2 avian influenza vaccine (CH9V) also elicited higher H9–Ag-specific IgG and IgG1 in mice and HI antibody titer in chicken, this robust humoral response was later produced. Moreover, serum IgG2a and IgG2b antibody titers in CH9V-immunized mice were significantly lower than those of IH9V alone group.

**Conclusions:**

AJSAF could improve antigen-specific humoral and cellular immune responses, and simultaneously trigger a Th1/Th2 response to IH9V. AJSAF might be a safe and efficacious adjuvant candidate for H9N2 avian influenza vaccine.

**Supplementary Information:**

The online version contains supplementary material available at 10.1186/s12917-020-02648-1.

## Background

H9N2 Low pathogenic avian influenza viruses (LPAIV) have been prevalent in multiple avian species worldwide [1–3], which has resulted in the tangible economic losses in poultry [4–6]. More importantly, H9N2 LPAIV from poultry has been reported to have human virus-like receptor specificity [7], resulting in public health concerns about the increase in pandemic potential in human [8–10]. The phylogenetic analysis of H7N9 influenza avian virus causing human respiratory infections revealed its six internal genes from chicken H9N2 LPAIV [11, 12]. H9N2 LPAIV was also proved to be the gene donor for H7N9 and H10N8 viruses infecting humans [13]. Recently, some cases of human infection with H9N2 LPAIV in China have been successively reported [14, 15]. Therefore, the eradication of H9N2 LPAIV in poultry becomes more important.

Vaccination is an economic and effective means for preventing and controlling H9N2 LPAIV in poultry. H9N2 LPAIV commercial vaccines consist mainly of the inactivated whole virus, requiring adjuvant for potentiating immunogenicity. Although many adjuvants were evaluated [16–19, 20], the current commercial H9N2 LPAIV vaccines cannot provide satisfactory protection against antigenically variant viruses [21, 22]. An ideal adjuvant will not only promote specific immune response, but also improve the type of immune response [23]. However, there were limitations in Th1 or Th2 biased responses generated by commercial H9N2 LPAIV vaccines.

In our previous studies, the total saponin from the stem bark of *A. julibrissin* and its purified active fraction AJSAF were reported to improve immune responses to ovalbumin, recombinant fowl pox virus vector-based H5 avian influenza vaccine [24], and porcine reproductive and respiratory syndrome virus (PRRSV) vaccine [25] in mice. In this study, AJSAF was evaluated for the adjuvant potentials on immune responses to inactivated H9N2 avian influenza virus vaccine (IH9V) in mice and chicken in comparison with commercially oil-adjuvant.

## Results

### Effect on the antigen-specific antibody response in immunized mice

**Fig. 1:**
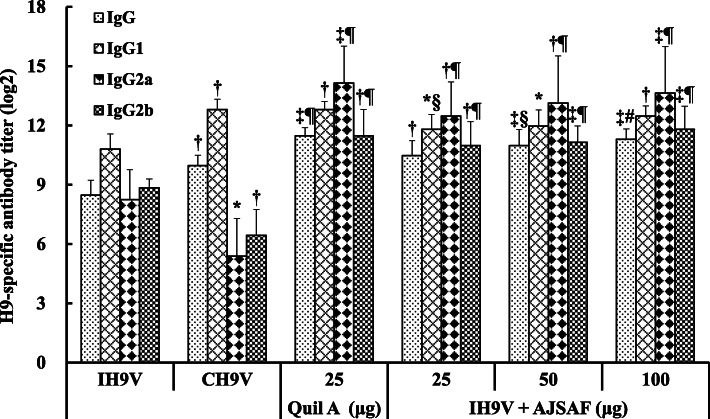
Effect of AJSAF on H9–Ag-specific IgG and its isotype antibody titers in the immunized mice. Mice were *s.c.* injected with 0.2 ml of IH9V (10^7^ TCID_50_/0.1 ml) alone or containing Quil A or AJSAF, or 0.2 ml of CH9V (10^7^ TCID_50_/0.1 ml) on days 1 and 15. Sera were collected 14 days after the boosting immunization, and serum H9–Ag-specific IgG, IgG1, IgG2a, and IgG2b antibody titers were measured by ELISA. The values are presented as means ± SD (*n* = 6). Significant differences with IH9V alone group were designated as ^*^P < 0.05, ^†^*P* < 0.01, and ^‡^*P* < 0.001; those with CH9V group as ^§^*P* < 0.05, ^#^*P* < 0.01, and ^¶^*P* < 0.001

**Fig. 2:**
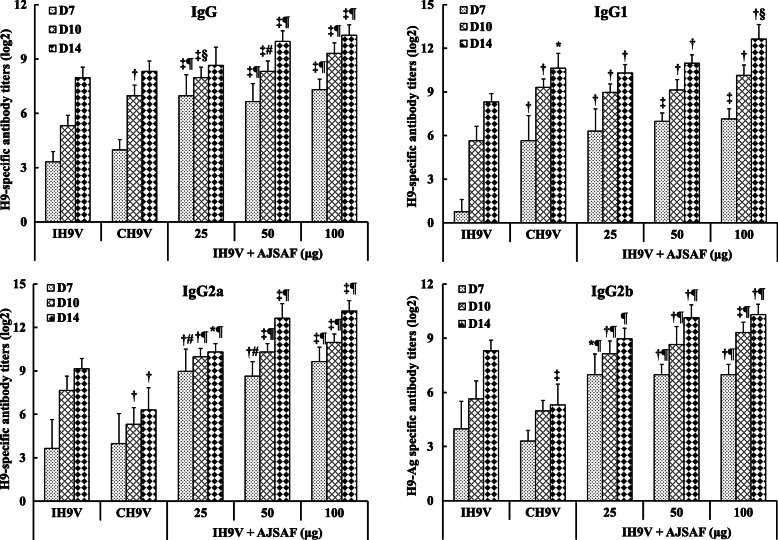
Time-response relationship of H9–Ag-specific IgG and its isotypes in the IH9V-immunized mice induced by AJSAF. Mice were *s.c.* immunized with 0.2 ml of IH9V (10^7^ TCID_50_/0.1 ml) alone or containing AJSAF, or 0.2 ml of CH9V (10^7^ TCID_50_/0.1 ml). Sera were collected at designated time after a single dose of vaccination for measuring H9–Ag-specific IgG, IgG1, IgG2a, and IgG2b antibody titers. The values are presented as means ± SD (*n* = 5). Significant differences with IH9V alone group were designated as ^*^P < 0.05, ^†^*P* < 0.01, and ^‡^*P* < 0.001; those with CH9V group as ^§^*P* < 0.05, ^#^*P* < 0.01, and ^¶^*P* < 0.001

As shown in Fig. 1. AJSAF and Quil A significantly raised serum H9–Ag-specific IgG, IgG1, IgG2a, and IgG2b antibody titers in IH9V-immunized mice (*P* < 0.05, *P* < 0.01, or *P* < 0.001). However, the serum IgG2a and IgG2b antibody titers in the mice immunized with the commercial oil-formulated inactivated H9N2 avian influenza vaccine (CH9V) were significantly lower than those in the mice immunized with IH9V alone (*P* < 0.05 or *P* < 0.01). Meanwhile, the serum H9–Ag-specific IgG, IgG1, IgG2a, and IgG2b antibody titers in the IH9V-immunized mice were significantly enhanced by AJSAF at 7, 10 and 14 days after a single dose of vaccination (*P* < 0.05, *P* < 0.01, or *P* < 0.001; Fig. 2). In contrast, oil adjuvant only significantly enhanced H9–Ag-specific IgG1 antibody titers at these three time points and IgG antibody titers 10 days after the immunization in the IH9V-immunized mice, respectively. Moreover, H9–Ag-specific IgG2a and IgG2b antibody titers in the CH9V-immunized mice with were significantly lower than those in mice immunized with IH9V alone at 14 days after the immunization.

### Effect on HI and H9–Ag-specific IgY antibody levels in immunized chicken

**Fig. 3:**
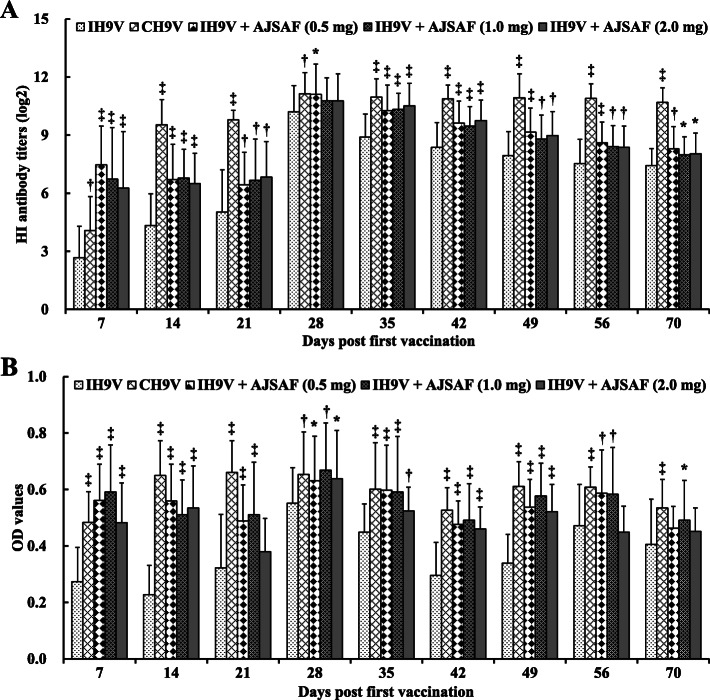
Effect of AJSAF on haemagglutination inhibition (HI) and H9–Ag-specific IgY antibody levels in the immunized chicken. The chickens were prime- and boost-immunized with 0.4 ml of IH9V (10^7^ TCID_50_/0.1 ml) alone or containing AJSAF, or 0.4 ml of CH9V (10^7^ TCID_50_/0.1 ml) at 3-week interval. Sera were collected on designated days post-immunization, and the serum HI antibody titers (**a**) and H9–Ag-specific IgY antibody (**b**) levels were measured by HI assay and ELISA, respectively. The values are presented as means ± SD (*n* = 30). Significant differences with IH9V alone group were designated as ^*^P < 0.05, ^†^*P* < 0.01, and ^‡^*P* < 0.001

The chickens were prime- and boost-immunized with IH9V alone or containing AJSAF, or CH9V at 3-week interval. As shown in Fig. 3a, IH9V alone induced lower serum haemagglutination inhibition (HI) antibody titers in chicken after the first immunization. HI antibody titers in chickens immunized with IH9V alone reaches to peak 1 week after a boosting immunization, and after that gradually decreased. AJSAF significantly enhanced the serum HI antibody titers in IH9V-immunized chickens at all the designated time points. The serum IgY antibody levels in chicken immunized with IH9V/AJSAF and CH9V were also significantly higher than those in chicken immunized with IH9V alone at all the designated time points (Fig. 3b).

### Effect on splenocyte proliferation in immunized mice

**Fig. 4:**
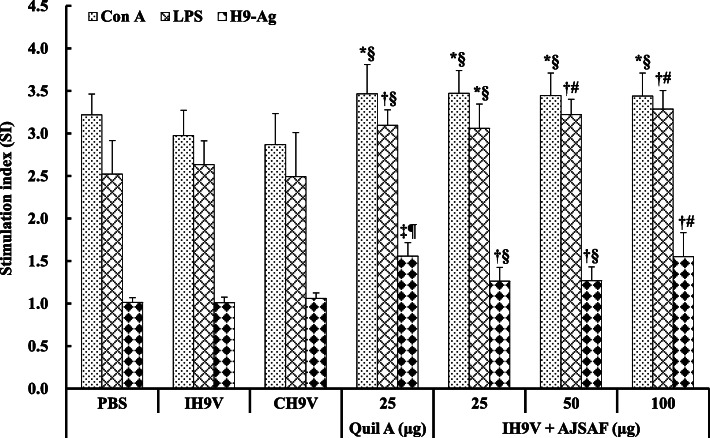
Effect of AJSAF on Con-, LPS-, and H9–Ag-stimulated splenocyte proliferation in the immunized mice. Splenocyte proliferation was measured by the MTT method and shown as a stimulation index (SI). The values are presented as means ± SD (*n* = 6). Significant differences with IH9V alone group were designated as ^*^P < 0.05, ^†^*P* < 0.01, and ^‡^*P* < 0.001; those with CH9V group as ^§^*P* < 0.05, ^#^*P* < 0.01, and ^¶^*P* < 0.001

Splenocyte proliferation was determined using 3-(4,5-dimethylthiazol-2-yl)-2,5-diphenyl- tetrazolium bromide (MTT) method, and the results are shown in Fig. 4. Con A-, LPS-, and H9–Ag-stimulated splenocyte proliferation in the mice immunized with IH9V/AJSAF and IH9V/Quil A were markedly higher than those in IH9V alone group (*P* < 0.05, *P* < 0.01, or *P* < 0.001). There were, however, no significant differences in Con A-, LPS- and H9–Ag-stimulated splenocyte proliferation between the IH9V alone and CH9V groups (*P* > 0.05).

### Effects on the activity of NK cells in immunized mice

**Fig. 5:**
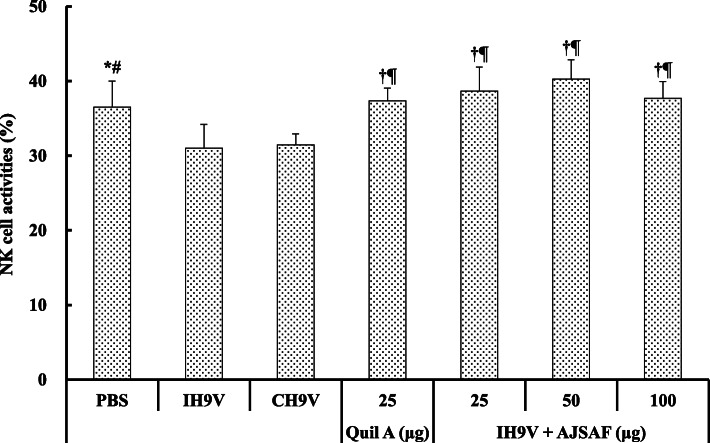
Effect of AJSAF on NK cell activity in the splenocytes from the immunized mice. Splenocytes were prepared 2 weeks after the boosting immunization for detecting NK cell activity by the MTT assay. The values are presented as means ± SD (*n* = 6). Significant differences with IH9V alone group were designated as ^†^*P* < 0.01and ^‡^*P* < 0.001; those with CH9V group as ^¶^*P* < 0.001

As shown in Fig. 5, the immunization with IH9V alone and CH9V resulted in the decrease in the killing activity of NK cells in mice (*P* < 0.05 or *P* < 0.01). There was no significant difference in NK cell activities was observed between the IH9V alone and CH9V groups (*P* > 0.05). However, AJSAF and Quil A significantly promoted the killing activity of NK cells from the splenocytes in the IH9V-immunized mice (*P* < 0.01 or *P* < 0.001).

### Effect on the secretion of cytokine from the splenocytes in immunized mice

**Fig. 6:**
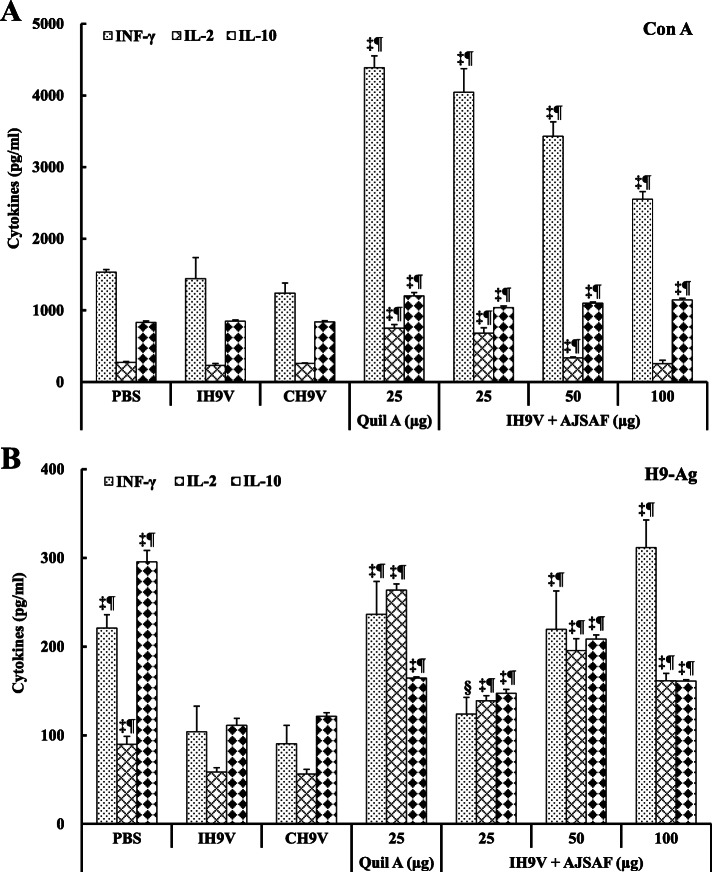
Effect of AJSAF on cytokine secretion from splenocytes in the immunized mice. Splenocytes were incubated with Con A (48 h) or H9–Ag (72 h), and the supernatants were collected for detecting IL-2, IFN-γ, and IL-10 levels using ELISA kits. The values are presented as means ± SD (*n* = 6). Significant differences with IH9V alone group were designated as ^*^P < 0.05, ^†^*P* < 0.01, and ^‡^*P* < 0.001; those with CH9V group as ^#^*P* < 0.01 and ^¶^*P* < 0.001

As shown in Fig. 6, AJSAF and Quil A significantly promoted the production of IL-2, IFN-γ, and IL-10 from Con A- and H9–Ag-stimulated splenocytes of the IH9V-immunized mice compared with IH9V alone group (*P* < 0.05, *P* < 0.01, or *P* < 0.001). There were, however, no significant differences were found in the production of IL-2, IFN-γ, and IL-10 from Con A- and H9–Ag-stimulated splenocytes between IH9V alone and CH9V groups (*P* > 0.05). In this experiment, it was also found that the contents of cytokines IL-2, IFN-γ, and IL-0 in the culture supernatants from H9–Ag-stimulated splenocytes in the mice immunized with IH9V alone and CH9V were significantly lower than those in PBS control mice (*P* < 0.01or *P* < 0.001; Fig. 6b).

### Effect on the mRNA expression levels of cytokines and transcription factors in splenocytes from immunized mice

**Fig. 7:**
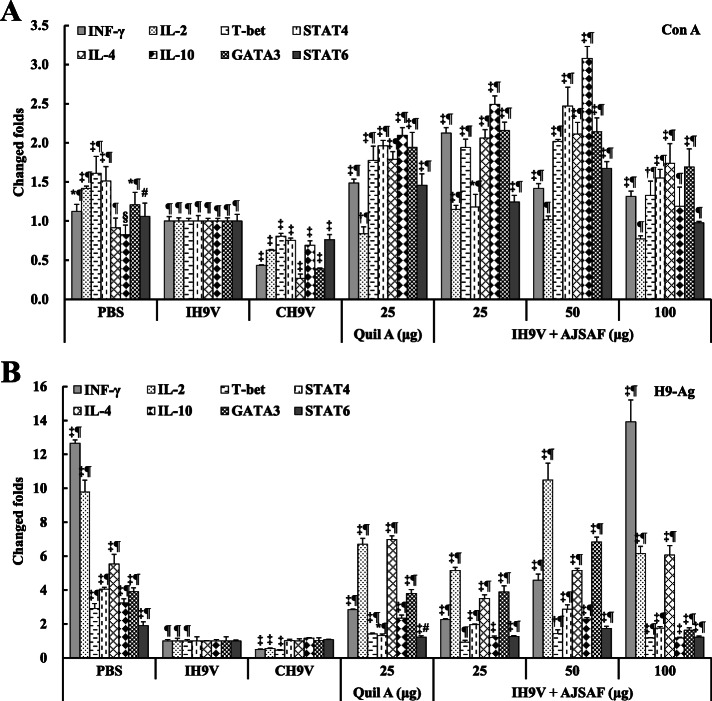
Effect of AJSAF on mRNA expression of cytokines and transcription factors in Con A (**a**) - and H9–Ag (**b**)-stimulated splenocytes from the immunized mice. Splenocytes were incubated with Con A (for 12 h) or H9–Ag (for 16 h), and the mRNA expression levels of cytokines and transcription factors were measured using RT-qPCR. The values are presented as means ± SD (*n* = 6). Significant differences with IH9V alone group were designated as ^*^P < 0.05, ^†^*P* < 0.01, and ^‡^*P* < 0.001; those with CH9V group as ^§^*P* < 0.05, ^#^*P* < 0.01, and ^¶^*P* < 0.001

As shown in Fig. 7, both AJSAF and Quil A significantly up-regulated not only the mRNA expression levels of Th1 (IL-2 and IFN-γ) and Th2 (IL-4 and IL-10) cytokines, but those of Th1 (T-bet and STAT-4) and Th2 (GATA3 and STAT-6) transcription factors in Con A- and H9–Ag-stimulated splenocytes from the mice immunized with IH9V (*P* < 0.05, *P* < 0.01, or *P* < 0.001). However, the immunization with IH9V alone and CH9V significantly down-regulated the mRNA expression levels of most cytokines and transcription factors in Con A- and H9–Ag-stimulated splenocytes of the mice compared to the PBS control mice (*P* < 0.05, *P* < 0.01, or *P* < 0.001).

## Discussion

Vaccination is an important and effective means of controlling H9N2 LPAIV in poultry. However, H9N2 LPAIV underwent significant antigenic drift [26], resulting in that the current commercially available vaccines cannot provide satisfactory protection [3, 22]. In contrast to B cells that have a subtype- and strains-specific response, the recognition and responses of T cell to influenza virus do not degrade with antigenic drift. It was reported that T cell responses were better correlated to influenza vaccine protection than antibody titers in human [27]. An optimal influenza vaccine should be capable of eliciting cell-mediated immunity as well as producing effective antibodies [28]. Consequently, many efforts have been made to develop novel efficacious adjuvants for inactivated LPAIV vaccine [29–33, 34].

Pedersen et al. [35] reported that the serum virus-specific IgG antibody titers correlated with influenza H5N1 virus neutralization in human. It was also reported that the induction of both IgG1 and IgG2a isotypes was considered to be a better correlate for vaccine efficacy than neutralization in mice [36]. In this study, AJSAF and Quil A significantly raised serum H9–Ag-specific IgG, IgG1, IgG2a, and IgG2b antibody titers in the IH9V-immunized mice (Fig. 1). However, the serum IgG2a and IgG2b antibody titers in the CH9V-immunized mice were significantly lower than those in the mice immunized with IH9V alone (*P* < 0.05 or *P* < 0.01). Meanwhile, the adjuvanticity–time relationship of AJSAF on the humoral immune responses to IH9V in mice was also investigated. The results showed that AJSAF could accelerate the humoral immune responses to IH9V in mice (Fig. 2).

To evaluate the adjuvant effect of AJSAF on immune responses to IH9V and profile the detailed dynamics of the antigen-specific humoral responses, serum HI and H9–Ag-specific IgY antibody in the immunized chicken were determined. The results showed that AJSAF significantly increased serum both HI antibody titers and H9–Ag-specific IgY levels in IH9V-immunized chickens up to 6 weeks post boosting immunization (Fig. 3). These results suggested that AJSAF could elevate and prolong HI antibody titers and H9–Ag-specific IgY antibody levels in immunized chicken. Interestingly, it was found that AJSAF could produce faster and approximately mean 18- and 7-fold higher antibody response in the chickens than the IH9V alone and CH9V 7 days after the first immunization, respectively. This finding is consistent with the time–course results of H9–Ag-specific IgG and its isotype response to IH9V in mice (Fig. 2), confirming that AJSAF could accelerate the humoral immune responses to IH9V.

The cellular immunity plays an important role in fighting influenza virus infections by limiting virus replication and accelerating clearance of virus-infected cells in mice [37]. The cross-protection responses mediated by influenza virus specific T cells directed not only towards conserved proteins such as nucleoprotein, but also to conserved sequences in hemagglutinin in mice [38]. To clarify the capacity of AJSAF to elicit effective T- and B-lymphocyte immunity, the Con A-, LPS-, and H9–Ag-stimulated splenocyte proliferation in the immunized mice was detected. The proliferation assay showed that AJSAF significantly promoted Con A-, LPS-, and H9–Ag-stimulated splenocyte proliferation in the IH9V-immunized mice. AJSAF was more effective than oil adjuvant in inducing strong activation potential of T and B cells, and could induce the humoral immunity and cell-mediated immune response to IH9V in mice (Fig. 4). In this investigation, the addition of oil-adjuvant to IH9V did also not result in increase in the killing activity of NK cells in the immunized mice. In contrast, AJSAF significantly promoted the NK cell activity in IH9V-immunized mice (Fig. 5), suggesting that the usage of AJSAF in IH9V could help to improve cytolytic activities against H9N2 LPAIV. These results suggested that AJSAF could elicit the cellular immune responses to IH9V in mice.

In order to clearly investigate that Th cell-derived cytokine profiles in IH9V-immunized mice, the contents of Th1 (IL-2 and IFN-γ) and Th2 (IL-10) cytokines in the culture supernatant of Con A- and H9–Ag-stimulated splenocytes were detected using ELISA kits. AJSAF significantly induced the production of IFN-γ, IL-2, and IL-10 from splenocytes in IH9V-immunized mice responding to Con A and H9–Ag (Fig. 6), suggesting that AJSAF could improve cell-mediated immunity and trigger dual Th1 and Th2 responses to IH9V.

To further elucidate the mechanism responsible for the efficacy of AJSAF on the Th1 and Th2 responses, the mRNA expression levels of cytokines and transcription factors in Con A- and H9–Ag-stimulated splenocytes from immunized mice were measured using RT-qPCR. AJSAF up-regulated the mRNA expression levels of not only Th2 cytokines (IL-4 and IL-10) and transcription factors (GATA3 and STAT-6), but also Th1 cytokines (IFN-γ and IL-2) and transcription factors (T-bet and STAT-4) (Fig. 7). The high mRNA expression of Th1 cytokines and transcription factors in splenocytes was consistent with the high IgG2a and IgG2b titers, while the high mRNA expression of Th2 cytokines and transcription factors was consistent with the high levels of IgG1 antibodies in mice immunized with IH9V/AJSAF [39]. Together, these results further confirmed that AJSAF improved the quality of the immune responses and elicited both Th1 and Th2 immune response to IH9V.

In addition, our experiment results also demonstrated that the immunization with IH9V and CH9V, especially the latter, impaired the cell-mediated immunity in mice, highlighting the crucial role of the adjuvant with enhanced cell-mediated immunity in effective vaccines against H9N2 LPAIV.

## Conclusions

In summary, this investigation demonstrated that AJSAF had immunological adjuvant activity on specific cellular and humoral immune responses and triggered both Th1 and Th2 responses to IH9V in mice. Most importantly, AJSAF could produce faster and higher HI antibody and virus-specific IgY response in chicken as well as antigen-specific antibody titers in mice. Hence, AJSAF may be an efficient adjuvant candidate for IH9V. This study also suggested that AJSAF-primed and oil-boosted regimen might produce encouraging results associated with quick, persistent and improved immune responses to inactivated H9N2 avian influenza vaccine, and be an economical and effective strategy for H9N2 LPAI prevention in poultry. This strategy and the clinical protective efficacy of AJSAF combined with IH9V on H9N2 LPAI needs to be further evaluated by the challenge test in chickens.

## Methods

### Materials

Concanavalin A (Con A), lipopolysaccharide (LPS), rabbit anti-mouse IgG peroxidase conjugate, RPMI medium, and 3-(4,5-dimethylthiazol-2-yl)-2,5-diphenyl-tetrazolium bromide (MTT) were purchased from Sigma Co., St. Louis, MO, USA; fetal calf serum was from Hyclone, Utah, USA; goat anti-mouse IgG1, IgG2a, and IgG2b peroxidase-conjugates were from Southern Biotech. Assoc., Birmingham, AL, USA; rabbit anti-chicken IgY horseradish peroxidase conjugate was from Promega Corporation, Madison, WI, USA; mouse cytokine detecting ELISA kits were from Wuhan Boster Bio-Tech. Co. Ltd., China; Trizol was from Invitrogen, Carlsbad, CA, USA; FastStart universal SYBR Green Master (ROX) was from Roche Diagnostics, Indianapolis, IN, USA. Quil A was gifted by Brenntag Nordic A/S, Hellerup, Denmark.

### Vaccines and viruses

The formaldehyde-inactivated H9N2 avian influenza virus (A/Chicken/Shanghai/1/98 [AIV H9N2 subtype, F Strain]) (IH9V) and its commercially oil-formulated vaccine (CH9V) were provided with Zhejiang CEVA EBVAC Biotech Co., Ltd., Hangzhou, China. IH9V and CH9V belong to the same lot (14008P) and both contain 10^7^ 50% tissue culture infective doses (TCID_50_)/0.1 ml. H9 subtype avian influenza virus antigen (H9–Ag), positive and negative serum were purchased from Beijing Kangnongxinmu Technology Development Center, Beijing, China.

### Preparation and characterization of AJSAF

The purified fraction of *Albizia julibrissin* saponins (AJSAF) was prepared and characterized as previously described [25]. A total of 29 saponins including 10 new compounds and 5 first found saponins from *A. julibrissin* were identified and characterized in AJASF by high-performance liquid chromatography coupled with quadrupole time-of-flight mass spectrometry based on accurate mass database [40].

### Experimental animals and immunization

Female ICR mice aged 5–6 weeks were purchased from Shanghai Experimental Animal Center of Chinese Academy of Sciences, Shanghai, China (certificate no. SCXK 2007–0005). SPF white Leghorn chickens were provided by Hangzhou Laying Hen Farm, Zhejiang, China. Animals were acclimatized for 1 week prior to use. Rodent laboratory chow and tap water were provided ad libitum and maintained under controlled conditions with a temperature of 24 ± 1 °C, humidity of 50 ± 10%, and a 12/12-h light/dark cycle.

In the experiment to evaluate the adjuvant effects of AJSAF on IH9V, mice were divided into seven groups, with six mice per group. Mice were subcutaneously (*s.c*.) injected with 0.2 ml of IH9V (10^7^ TCID_50_/0.1 ml) alone or containing Quil A (10 μg), or AJSAF (25, 50, or 100 μg) on day 1. A boosting injection was given 14 days later. Animals injected with 0.2 ml of PBS and CH9V (10^7^ TCID_50_/0.1 ml) were included as a negative and positive control, respectively. Sera and splenocytes were collected Two weeks after the boosting immunization.

In adjuvanticity–time analysis experiment, mice were divided into six groups of fifteen mice each. Mice were *s.c.* immunized with 0.2 ml of IH9V (10^7^ TCID_50_/0.1 ml) alone or containing AJSAF (25, 50, or 100 μg), or 0.2 ml of CH9V (10^7^ TCID_50_/0.1 ml) on day 1. Saline-treated mice were included as controls. Five mice per group were randomly sacrificed at 7, 10, and 14 days after the immunization, and sera were collected for measurement of H9–Ag-specific antibody titers. All the animals were euthanized in a chamber with carbon dioxide. Blood was drawn from the eyes and the serum was prepared by centrifuging at 3500 rpm for 10 min at 4 °C. The mice were then sacrificed via cervical dislocation, and the spleens were collected.

35-day-old SPF white Leghorn chickens were divided into five groups of 30 chickens each, and intramuscularly (*i.m.*) immunized with 0.4 ml of IH9V (10^7^ TCID_50_/0.1 ml) alone or containing AJSAF (0.5, 1.0, or 2.0 mg), or 0.4 ml of CH9V (10^7^ TCID_50_/0.1 ml) on day 1. A boosting injection was given 3 weeks later. Sera were collected at 1- or 2-week intervals for measurement of antibody. Finally, all chickens were euthanized by intravenous injection with pentobarbital sodium at the dose 40 mg/kg followed by cervical dislocation.

### Measurement of serum H9–Ag-specific antibody

Serum H9–Ag-specific IgG antibody and its isotypes in mice and IgY antibody in chicken were detected by an indirect ELISA as previously described [41]. The optical density was measured at 492 nm using a BIO-RAD 680 ELISA reader. The mean OD value of the sample was subtracted from that of the control. Where sets of serum samples have been subjected to within and between group comparisons, ELISA assays were performed on the same day for all of the samples.

### Haemagglutination inhibition (HI) assay

The serum inactivated at 56 °C for 30 min was two-fold serially diluted with PBS. The diluted serum was added to 96-well V-bottom plates, and then incubated with an equal volume of H9–Ag (4 hemagglutinating units (HAU)) for 30 min at room temperature. An equal volume of 1% chicken red blood cells were added, and the plates were incubated for further 30 min. The positive and negative sera were included as controls. The HI titers were represented as the log2 of the reciprocal of the highest serum dilution leading to complete HI [17].

### Splenocyte proliferation assay

Splenocytes (5 × 10^6^ cells/ml) were incubated with Con A (5 μg/ml), LPS (10 μg/ml), H9–Ag (0.125 HAU/ml), or medium in 96-well culture plate at 37 °C and 5% CO_2_. After 44 h (Con A and LPS) or 68 h (H9–Ag), splenocyte proliferation was detected using MTT assay as previously described [40], and shown as the stimulation index (SI).

### Assay of NK cell activity

The activity of NK cells in splenocytes was measured by MTT assay using human leukemia K562 cell lines as target cells as previously described [42]. Three kinds of control measurements were performed: target cells control, blank control and effector cells control. NK cell activity was calculated as following equation: NK activity (%) = (OD_T_ − (OD_S_ − OD_E_))/OD_T_ × 100, where OD_T_, absorbance of target cells control, OD_S_, absorbance of test samples and OD_E_, absorbance of effector cells control.

### Cytokine measurements

Splenocytes (5 × 10^6^ cells/well) were incubated with Con A (5 μg/ml) and H9–Ag (0.125 HAU/ml) in 24-well culture plates at 37 °C in 5% CO_2_ for 48 and 72 h, respectively. The supernatants were collected for detecting contents of cytokines (IL-2, IL-10, and IFN-γ) using ELISA kits [43].

### Real-time quantitative PCR (RT-qPCR)

Splenocytes were incubated with Con A (2.5 μg/ml) and H9–Ag (0.125 HAU/ml) in 24-well culture plates for 12 and 16 h at 37 °C in 5% CO_2_, respectively. The cells were harvested and subjected to Trizol reagent to isolate total RNA. The reverse transcription was conducted as previously described [44]. The qPCR was performed on an ABI PRISM® 7300 PCR System using FastStart Universal SYBR Green Master. The sequences of primers for qPCR were listed in Table S1. The qPCR cycling was performed as follows: one cycle at 95 °C for 10 min, followed by 40 cycles of denaturation at 95 °C for 15 s, and then annealing at 60 °C for 1 min. The mRNA expression levels of the tested genes relative to GAPDH were determined using the 2^-ΔΔCt^ method and as fold induction.

### Statistical analysis

Data were expressed as mean ± SD and the statistical significance of difference was analyzed with ANOVA and a Tukey post-hoc test. *P*-value less than 0.05 was considered to be statistically significant.

## Supplementary Information

**Additional file 1:** **Table S1.** Primer used for RT-qPCR.

## Data Availability

The data analyzed during the current study are are included in this published article.

## Abbreviations

AJSAF: Purified active saponin fraction from the dry stem bark of *Albizzia julibrissin*
CH9V: Commercial oil-formulated H9N2 avian influenza vaccine
Con A: Concanavalin A
ELISA: Enzyme-linked immunosorbent assay
H9–Ag: H9 subtype avian influenza viruses antigen
HAU: Hemagglutinating unit
HI: Haemagglutination inhibition
IH9V: Inactivated H9N2 avian influenza virus vaccine
LPAIV: Low pathogenic avian influenza virus
LPS: lipopolysaccharide
MTT: 3-(4,5-dimethylthiazol-2-yl)-2,5-diphenyl-tetrazolium bromide
NK cell: Natural killer cell
RT-qPCR: real-time quantitative polymerase chain reaction
Quil A: Total saponin from *Quillaja saponaria* Molina
TCID_50_: 50% tissue culture infective doses
