# Supplementary material for: Quick and improved immune responses to inactivated H9N2 avian influenza vaccine by purified active fraction of Albizia julibrissin saponins
Source: BMC Vet Res. 2020 Nov 7;16:427. doi: 10.1186/s12917-020-02648-1 (PMC7648552; doi:10.1186/s12917-020-02648-1)
Supplement: Supplementary file 1 — Additional file 1: Table S1. Primer used for RT-qPCR. [file 12917_2020_2648_MOESM1_ESM.doc]

**Table S1.** Primer used for qRT-PCR.

| Gene | Primer sequence | Product size (bp) |
| --- | --- | --- |
| GAPDH | 5’-AGCCTCGTCCCGTAGACAA-3’  5’-AATCTCCACTTTGCCACTGC-3’ | 104 |
| IL-2 | 5’-CCCAAGCAGGCCACAGAATTGAAA-3’  5’-AGTCAAATCCAGAACATGCCGCAG-3’ | 81 |
| IFN-γ | 5’- TCTTGAAAGACAATCAGGCCATCA -3’  5’- GAATCAGCAGCGACTCCTTTTCC -3’ | 233 |
| IL-4 | 5’-CAAACGTCCTCACAGCAACG-3’  5’-CTTGGACTCATTCATGGTGC-3’ | 203 |
| IL-10 | 5’- GCTCTTACTGACTGGCATGAG -3’  5’- CGCAGCTCTAGGAGCATGTG -3’ | 105 |
| T-bet | 5’-GATCATCACTAAGCAAGGACGGC-3’  5’-AGACCACATCCACAAACATCCTG-3’ | 101 |
| GATA3 | 5’-AGTCCTCATCTCTTCACCTTCC-3’  5’-GGCACTCTTTCTCATCTTGCCT-3’ | 112 |
| STAT-4 | 5’-TGGCAACAATTCTGCTTCAAAAC-3’  5’-GAGGTCCCTGGATAGGCATGT-3’ | 225 |
| STAT-6 | 5’-CTCTGTGGGGCCTAATTTCCA-3’  5’-CATCTGAACCGACCAGGAACT-3’ | 135 |

GAPDH, glyceraldehyde-3-phosphate dehydrogenase.
